# Supplementary material for: The interactome of intact mitochondria by cross-linking mass spectrometry provides evidence for coexisting respiratory supercomplexes
Source: Mol Cell Proteomics. 2017 Dec 8;17(2):216–32. doi: 10.1074/mcp.RA117.000470 (PMC5795388; doi:10.1074/mcp.RA117.000470)
Supplement: Supplemental Data [file supp_RA117.000470_133922_0_supp_23978_fzffwf.zip › spectra_annotation/mito_DR_spectra_annotation/443-1-2-1-5-1.pdf]

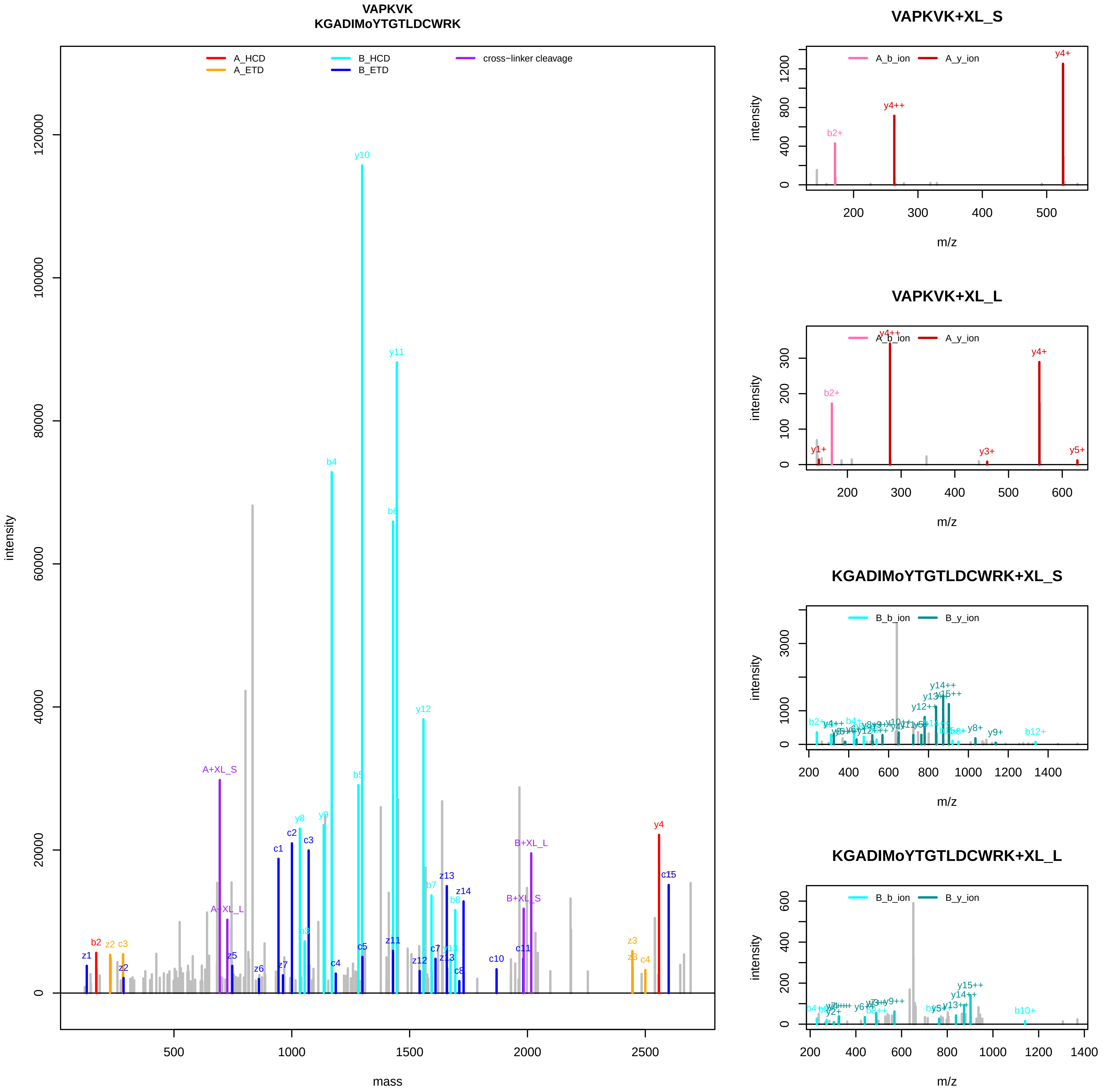

VAPKVK
KGADIMoYTGTLDCWRK
A_HCD
A_ETD
B_HCD
B_ETD
cross−linker cleavage
intensity
mass
0
20000
40000
60000
80000
100000
120000
500
1000
1500
2000
2500
y10
y11
b4
b6
y12
A+XL_S
A+XL_L
B+XL_L
B+XL_S
y4
c15
z1
b2
z2
c3
c1
c2
y8
y9
b5
z13
z14
c10
c11
z3
c4
VAPKVK+XL_S
A_b_ion
A_y_ion
y4+
y4++
b2+
m/z
VAPKVK+XL_L
y1+
y3+
y5+
KGADIMoYTGTLDCWRK+XL_S
B_b_ion
B_y_ion
y14++
y15++
y12++
y8+
y9+
b12+
KGADIMoYTGTLDCWRK+XL_L
y15++
y14++
b10+
